# Knowledge of Zika Virus Transmission and Its Prevention among High-Risk Pregnant Women in Brazil

**DOI:** 10.3390/v13020242

**Published:** 2021-02-04

**Authors:** Lucas C. Pires, Luiza R. Dantas, Steven S. Witkin, Ana Paula A. P. Bertozzi, Rita de Cássia A. B. Dezena, Maria M. D. Rodrigues, Rosa Estela Gazeta, Saulo D. Passos

**Affiliations:** 1 Faculty of Medicine, Jundiaí School of Medicine, Jundiaí, São Paulo 13202-550, Brazil; luizadantas64@gmail.com; 2 Department of Obstetrics and Gynecology, Weill Cornell Medicine, New York, NY 10065, USA; switkin@med.cornell.edu; 3 Laboratory of Virology, Institute of Tropical Medicine, University of São Paulo, São Paulo 05403-000, Brazil; 4 Department of Pediatrics, Jundiaí School of Medicine, Jundiaí, São Paulo 13207-450, Brazil; bertozzianap@gmail.com (A.P.A.P.B.); rita10nurse@yahoo.com.br (R.d.C.A.B.D.); mmanoelarodrigues@bol.com.br (M.M.D.R.); regazeta@uol.com.br (R.E.G.); sauloduarte@uol.com.br (S.D.P.); 5 Laboratory of Pediatric Infectious Disease, Jundiaí School of Medicine, Jundiaí, São Paulo 13202-550, Brazil

**Keywords:** Zika virus, pregnancy, maternal health, infant health, disease prevention, public health

## Abstract

Recent outbreaks of Zika virus (ZIKV) infection highlight the urgent need to evaluate the efficacy of current public health measures to educate susceptible groups about how to prevent infection, modes of viral transmission, and consequences of infection. We performed a cross-sectional study in the city of Jundiaí, São-Paulo, from March 2016 to August 2017. In 315 high-risk pregnant women we evaluated the rate of ZIKV infection, knowledge of pathways of ZIKV transmission, and the use of protective measures. Data were analyzed and correlated with sociodemographic variables. The rate of ZIKV infection was 10.8%. ZIKV transmission by mosquitoes was the best-known means of virus acquisition, while transmission of ZIKV by sexual intercourse as well as mother–fetus transmission was known by less than half of the women. The use of insect repellent, reported by 53% of participants, was correlated with higher education and personal directives from health professionals. Condom use was reported by 19.5% of subjects. Improved strategies to increase awareness of ZIKV infection and its consequences, designed to appeal to specific, targeted populations, are clearly necessary to more accurately prevent the spread of this infection and diminish adverse consequences in the pregnant population.

## 1. Introduction

Vector-borne diseases account for more than 17% of all infections worldwide, killing more than 700,000 people per year. The vast majority of deaths can be prevented through protective measures and community mobilization [1]. The arboviruses (arthropod-borne viruses) are responsible for many devastating diseases in humans with public health importance, such as Zika virus (ZIKV), Yellow Fever, and West Nile viruses [2]. ZIKV, a member of the Flaviviridae family, was declared in 2016 by the World Health Organization as a Public Health Emergency of International Concern [3]. Despite the fact Brazil is not currently facing ZIKV epidemic, during the period of December 2019 to October 2020, there were 7000 suspected cases, with a total of 201 confirmed cases during pregnancy in Brazil [4].

A unique characteristic of ZIKV is its diversity in terms of transmission. Infection is primarily from mosquito bites but can also occur by sexual intercourse [5], blood transfusion, accidental blood exposure, transplacentally [6,7], and possibly via breast milk [8]. ZIKV has been isolated from amniotic fluid, cerebrospinal fluid, urine, saliva, vaginal secretions, semen, and breast milk [9].

The symptoms of ZIKV infection are nonspecific, and the majority of patients are asymptomatic [2]. This makes accurate diagnosis and patient follow-up difficult [10]. During pregnancy, ZIKV infection may induce Congenital Zika Syndrome (CZS) in fetuses and newborns, and ZIKV is now a member of the TORCH group (Toxoplasmosis, Other, Rubella, Cytomegalovirus, Herpes Simplex Virus) [11]. CZS can present with a large spectrum of neurological and ortho-muscular changes, such as brain calcification, ventriculomegaly, joint and ocular abnormalities, and microcephaly [12]. Some infected newborns may present developmental delay and neurosensory alterations during the first years of life, showing that even when CZS is not diagnosed at birth, exposed babies may, nevertheless, experience long-term consequences of intrauterine exposition to ZIKV [13]. These findings affect the quality of life of the entire family in terms of psychological, medical, and social consequences [14]. In addition, there are severe economic consequences.

Identification of effective strategies to prevent ZIKV transmission, especially to pregnant women, is an obvious priority [15]. Since there is no vaccine or specific treatment for ZIKV, prevention and education are key factors in controlling and combating this infection [2,16,17,18]. Surveying a population’s level of knowledge about ZIKV, its outcomes, and ways of prevention are essential for the design and adoption of successful intervention strategies [19]. However, studies assessing information and attitudes toward ZIKV infection are limited and identification of knowledge gaps can lead to decreased susceptibility to infection [20], especially in pregnant women. This group has the highest incidence of comorbidities that justifies an enhanced concern about ZIKV exposure during prenatal care and the subsequent increased risk of complications in both mother and newborn [12,21].

Utilizing a pregnant population at the time of a recent ZIKV epidemic, the present study, carried out between March 2016 and August 2017, evaluated the frequency of ZIKV infection and subjects’ knowledge of methods to limit virus exposure and the routes of ZIKV transmission. Findings were correlated with socio-demographic variables.

## 2. Materials and Methods

### 2.1. Study Area

The study was performed at the University Hospital of the Faculty of Medicine of Jundiaí, a regional center specializing in gynecology, obstetrics, and pediatrics. The municipality of Jundiaí has 423,000 inhabitants [22].

### 2.2. Study Design

We conducted a cross-sectional study, part of the Thematic Project: “Vertical infection by the ZIKA virus and its impact on the mother-child area”, a Cohort Study that recruited women with high-risk pregnancies between March 2016 and August 2017 [23,24]. The inclusion criteria were (1) high-risk pregnant women, defined as pregnancies more likely to have an adverse outcome due to previous pregnancy loss or preterm delivery, preexisting autoimmune, hormonal or hypertensive conditions, the presence of hormonal, infectious, or hypertensive disorders in the current pregnancy, or maternal age <16 or >35 [25], (2) participation in the Thematic Project, and (3) residents of Jundiaí. The exclusion criteria were women with life-threatening conditions and those unable to provide written, informed consent.

### 2.3. Study Instrument and Variables

**Figure 1:**
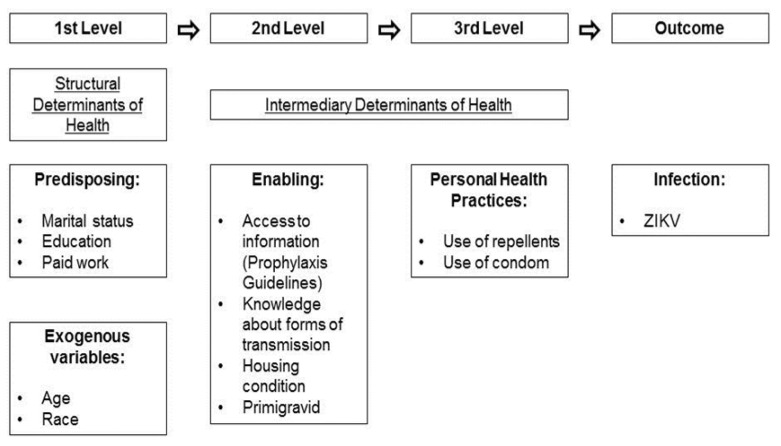
Conceptual Framework.

A pilot study was conducted starting in January 2017 with the objectives of training the team of researchers, evaluating subjects’ understanding of the questions, and estimating the duration of the interviews. This training lasted two months and each interview lasted an average of 25 min. After the pilot test, we finalized a semi-structured questionnaire, based on recommendations indicated by the World Health Organization (WHO) [26], with the conceptual framework adapted for the study [19,27] (Figure 1). The questionnaire evaluated the following aspects: (1) socio-demographic characteristics: age, race, marital status, educational level, paid work, and people per room in the household, (2) epidemiological and organizational variables: contact with other arboviruses, travel to possible risk areas, and receiving calls from the Cohort (for internal control and active search for complications), (3) prevention: use of condoms and insect repellent, (4) knowledge about ZIKV transmission routes (sexual, vertical, insect vector), and (5) guidelines on how to acquire the knowledge and means to prevent ZIKV acquisition (health professionals, television, internet, newspaper, radio, family, or friends).

The final questionnaire was given to 315 subjects from April 2017 to February 2018 by previously trained researchers during a scheduled prenatal visit of women in the Cohort. After application of the questionnaire, all subjects received ZIKV prevention guidelines and other pertinent recommendations about the disease, as well as free distribution of insect repellents and condoms.

### 2.4. Statistical Analysis

Collected data were entered into an Excel spreadsheet and analyzed through Statistical Package of Social Sciences (IBM SPSS Statistics 23, IBM Corporation, Armonk, NY, USA). Cases with missing data were automatically dropped from each analysis by SPSS. The dependent variables in the study were: orientation about ZIKV prevention, use of insect repellent, use of condoms, and presence of ZIKV infection. ZIKV infection was defined as detection of ZIKV RNA by RT-PCR or identification of specific IgM antibodies by ELISA, as described in the cohort profile [23]. After a descriptive analysis, the covariates were categorized and/or dichotomized for statistical analysis [28]. To compare dependent and covariates, data were analyzed by the Chi-square test or Fisher’s exact test as appropriate. Logistic regression analysis was used to determine the predictors of each dependent variable: ZIKV positivity, orientation of preventive measures, and repellent and condom use. We used univariate logistic regression, and variables with a *p* value <0.20 were entered into the multivariate analysis. The confidence interval adopted was 95%. The Hosmer and Lemeshow Test was used to verify adherence of the models, considered a poor fit if the *p* value was <0.05.

### 2.5. Ethical Approval

This study was approved in the domain of the Jundiaí Zika Cohort by the Research Ethics Committee of the Faculty of Medicine of Jundiaí (CAAE 53248616200005412, protocol number 1446577, 10 March 2016); Ethics and Research Review Committee (WHO ERC), World Health Organization (ZIKV100/2018). All research was conducted within ethical standards and the confidentiality of all information was ensured to maintain the privacy of the participants.

## 3. Results

### 3.1. Study Population Characteristics

Characteristics of study participants are shown in Table 1. Median age was 27.9 years (range: 13–46 years). Most of the subjects were White (52.1%) or mixed race (34.8%). Most (78.6%) were either married or in stable relationships and 41.5% had a high school education. Crowded housing conditions (≥1 person per room) were reported by 55.4% of subjects, 46.2% worked outside of the home, and 32.4% were having their first pregnancy.

### 3.2. Guidance about ZIKV Prevention

Information about ZIKV prevention and transmission and the sources of knowledge are displayed in Table 2. Knowledge about modes of ZIKV transmission was acknowledged by 85.9% of the subjects. Most (83.8%) knew that the virus was transmitted by mosquitoes, 42.8% were aware that ZIKV could be sexually transmitted, and 42.1% knew that a pregnant woman could transmit the virus to her unborn baby. The majority of the women obtained information about ZIKV either from a health professional (66.4%) or from television (59.4%).

The use of insect repellent was reported by 53% of participants. We identified an increased likelihood to use insect repellent if the participants had higher than an eighth-grade education (*p* = 0.014, adjusted Odds Ratio (aOR) 2.114, 95% Confidence Interval (CI) 1.162, 3.844) and had been personally instructed to do so by health professionals (*p* = 0.001, aOR 2.855, 95% CI 1.515, 5.380). The complete analysis is shown in Table S1. The Hosmer and Lemeshow Test yielded a *p* value of 0.875. Condom use during pregnancy was reported by 19.5% of the subjects, and absence of this use was associated with receiving ZIKV information via television (*p* = 0.049, aOR 0.493, 95% CI 0.244, 0.997). The complete analysis is shown in Table S2. The Hosmer and Lemeshow Test *p* value was 0.473.

### 3.3. ZIKV Infection

Thirty-four (10.8%) of the women were positive for ZIKV, 97.1% detected by RT-PCR and 2.9% by identification of specific IgM antibodies. The prevalence of ZIKV infection was similar among women regardless of differences in socioeconomic variables, their use of prophylactic measures, or knowledge about ZIKV transmission (Table 3). The complete analysis is shown in Table S3.

## 4. Discussion

The results of our study indicate that, even in the middle of a viral epidemic, the group most susceptible to adverse consequences, high-risk pregnant women, were seriously under-informed about the consequences of ZIKV infection and means of prevention. Use of insect repellent or condoms, receiving guidance on prevention, and knowledge about routes of transmission were reported by only a limited number of subjects, 10.8% of whom were ZIKV-positive. This highlights the inadequacy of current public health measures and the need for improved means of communication to the general public. Paralleling findings from other studies [29,30,31], the transmission of ZIKV by an insect vector was known to a greater number of subjects than were the perinatal and sexual transmission pathways. The use of repellents by approximately half of the participants was similar to previous studies of pregnant women conducted in Martinique (49.5%) [32] and Brazil (56%) [33] and different from what was found in the US Virgin Islands, where 74% of the pregnant women reported using repellent [34]. The use of repellents was associated with higher levels of education and receiving direct guidance from health professionals. This was also consistent with findings from prior studies [33,35,36]. This reinforces the need for comprehensive educational campaigns and widely available information [37], appropriate to the social cultural condition of target population [20,38].

While an enhanced access to information translates to a higher level of prevention [32,39], socioeconomic and cultural conditions are additional highly relevant variables that can directly reflect individuals’ attitudes and prevention practices [40]. The cost of insect repellent may be one of the obstacles to its use [33,35]. Programs that include free distribution of preventative items is an effective method in the control of infections, especially in populations with few resources, as has already been observed in a study from Puerto Rico [41]. However, in our study, repellents were freely available at no cost. Additional factors must, therefore, be involved in low adherence, such as the absence of frequent reinforcement of its importance and a low level of understanding of the consequences of infection [42].

The use of condoms to prevent transmission of ZIKV during sexual intercourse was infrequently used in our population. Consistent with prior studies [32,34,37,39], condom usage for a reason other than contraception is understandably unpopular. Being a participant in a stable union was associated with the absence of condom usage, repeating findings from another Brazilian study [43]. We also observed a weak association between non-use of condoms and receiving knowledge about ZIKV from television. Again, this is likely related to an aversion to recommending condoms to couples in which the woman is already pregnant.

Dissemination of information by television has a recognized role in reducing the risk of arbovirus infections in Brazil [44] and, in the present study, this was the second most common method of receiving prevention guidance. Wakefield et al. [45] recommended strategies that could improve the success of information transmission by television: a more frequent and more widespread exposure to information and messages based on an understanding of the types of presentation most likely to appeal to the target audience. An improved understanding of ZIKV-related knowledge and the means of acquisition in a specific population can assist in development and restructuring of public health policies to improve strategies for the prevention, combat, and surveillance of ZIKV infection [10,46].

Findings from this study should be viewed in light of several limitations. The study sample represented a specific population of high-risk pregnant women who were users of the public health system. Therefore, the results may not be generalizable to other populations. The data that we obtained were cross sectional, which did not allow for causal inference, and were based on self-report. This may have introduced measurement error from recall bias or social desirability.

## 5. Conclusions

Current public health measures to educate our high-risk pregnant population about the modes of ZIKV transmission and how to prevent exposure were clearly inadequate. The frequency of ZIKV infection was 10.8% and knowledge was very limited. This lack of understanding about ZIKV and insufficient practice of disease prevention indicates the need for continuous reassessment to improve educational strategies in public health. Novel and improved strategies to increase awareness of ZIKV infection and its consequences, designed to appeal to specific, targeted populations, are clearly necessary to more accurately educate and lower the incidence of ZIKV infection and its consequences in vulnerable groups.

## Figures and Tables

**Table 1:** Characteristics of total study population.

Characteristics of Pregnant Women	
Median age (range)	27.9 (13–46) years
Marital status	
Stable partnership	40.1%
Married	38.5%
Single	18.8%
Divorced	1.6%
Widowed	1.0%
Race	
White	52.1%
Mixed race	34.8%
Black	11.5%
Asian	1.0%
Indian	0.6%
Education	
Elementary school	4.1%
Middle school	18.9%
Some high school	18.8%
Completed high school	41.5%
Attended college	16.6%
Employed outside the home	46.2%
≥1 persons per room	55.4%
First pregnancy	32.4%

**Table 2:** Knowledge about ZIKV transmission.

Variable	Parameters	Values
Transmission		
	Insect vector	83.8%
	Perinatal	42.1%
	Sexual intercourse	42.8%
Source of knowledge		
	Health professional	66.4%
	Television	59.4%
	Internet	30.6%
	Newspapers	13.5%
	Friends or family	7.9%
	Radio	6.1%

**Table 3:** Association between variables and ZIKV infection.

Variable	Parameters	ZIKV Detection	
		Positive	Negative
Marital status			
	Stable union	82.4%	78.2%
	No stable partner	17.6%	21.8%
Age			
	<35	85.3%	79.7%
	≥35	14.7%	20.3%
Education			
	≤8 years	44.1%	41.6%
	>8 years	55.9%	58.4%
Race			
	White	55.9%	51.6%
	Other	44.1%	48.4%
Work outside the home			
	Yes	47.1%	46.0%
Persons per room			
	<1	39.4%	45.3%
	≥1	60.6%	54.7%
Primigravid		41.2%	31.3%
Insect repellent usage		64.7%	51.6%
Condom usage		17.6%	19.7%
Knowledge of ZIKV transmission *			
	Insect vector	93.1%	82.8%
	Perinatal	48.3%	41.4%
	Sexual intercourse	58.6%	41.0%
Source of knowledge *			
	Health professional	70.8%	65.9%
	Television	70.8%	58.0%
	Internet	41.7%	29.3%

* Column percentages do not total 100% because categories are not mutually exclusive.

## Data Availability

The data are contained within the article or Supplementary Materials.

## Supplementary Materials

The following are available online at https://www.mdpi.com/1999-4915/13/2/242/s1, Table S1: Univariate and multivariate logistic regression analysis showing associations between variables and use of insect repellent; Table S2: Univariate and multivariate logistic regression analysis showing associations between variables and use of condom; Table S3: Univariate and multivariate logistic regression analysis of associations between variables and ZIKV infection.
