# Supplementary material for: Knowledge of Zika Virus Transmission and Its Prevention among High-Risk Pregnant Women in Brazil
Source: Viruses. 2021 Feb 4;13(2):242. doi: 10.3390/v13020242 (PMC7913803; doi:10.3390/v13020242)
Supplement: Supplementary file 1 [file viruses-13-00242-s001.pdf]

viruses

MDPI

Supplementary Materials: Knowledge of Zika Virus Transmission and its Prevention among High–Risk Pregnant Women in Brazil

Table S1. Univariate and multivariate logistic regression analysis showing associations between variables and use of insect repellent.

Variables		All subjects		Repellent use									
				Yes		Not		*p*–value	OR	[CI 95%]	*p*–value	aOR	[CI 95%]
		n	%	n	%	n	%						
Marital status	Stable union	247	78.7	139	83.7	108	73.0	0.020*	1.907	1.101–3.302	0.059	1.940	0.976–3.854
	Not stable partner	67	21.3	27	16.3	40	27.0		Ref.			Ref.	
Age	< 35 years	253	80.3	131	78.4	122	82.4	0.374	Ref.				
	≥ 35 years	62	19.7	36	21.6	26	17.6		1.289	0.735–2.261			
Education	≤ 8 years	131	41.9	53	31.9	78	53.1	0.000*	Ref.		0.014*	Ref.	
	> 8 years	182	58.1	113	68.1	69	46.9		2.410	1.522–3.817		2.114	1.162–3.844
Race	White	163	52.1	92	55.8	71	48.0	0.169	1.367	0.876–2.134	0.228	1.436	0.798–2.584
	Other	150	47.9	73	44.2	77	52.0		Ref.			Ref.	
Work outside the home	Yes	144	46.2	87	52.7	57	38.8	0.014*	1.761	1.122–2.765	0.534	1.205	0.669–2.172
	Not	168	53.8	78	47.3	90	61.2		Ref.				
Person per room	<1	128	44.6	70	46.7	58	42.3	0.461	1.192	0.747–1.900			
	≥1	159	55.4	80	53.3	79	57.7		Ref.				
Knowledge of ZIKV transmission		290		149		141							
	Insect vector	243	83.8	129	86.6	114	80.9	0.186	1.528	0.813–2.870	0.326	2.697	0.372–19.555
	Perinatal	122	42.1	58	38.9	64	45.4	0.265	0.767	0.481–1.224			
	Sexual intercourse	124	42.8	72	48.3	52	36.9	0.049*	1.600	1.001–2.559	0.391	1.307	0.709–2.409
	None	41	14.1	17	11.4	24	17.0	0.170	0.628	0.321–1.226	0.291	3.304	0.360–30.356
Source of knowledge		229		119		110							
	Internet	70	30.6	38	31.9	32	29.1	0.641	1.144	0.651–2.009			
	Television	136	59.4	70	58.8	66	60	0.856	0.952	0.562–1.615			

	Health professional	152	66.4	92	77.3	60	54.5	0.000*	2.840	1606–5.021	0.001*	2.855	1.515–5.380
Primigravid	Yes	94	32.4	51	34.5	43	30.3	0.447	1.211	0.739–1.982			
	Not	196	67.7	97	65.5	99	69.7		Ref.				

*$p < 0.05$; CI: Confidence interval; aOR: adjusted Odds Ratio.

Table S2. Univariate and multivariate logistic regression analysis showing associations between variables and use of condom.

**Variables**		**All subjects**		**Condom use**				***p*–value**	**OR**	**[CI 95%]**	***p*–value**	**aOR**	**[CI 95%]**
				Yes		Not							
		n	%	n	%	n	%						
Marital status	Stable union	239	79.1	41	69.5	198	81.5	0.042*	Ref.		0.135	Ref.	
	Not stable partner	63	20.9	18	30.5	45	18.5		1.932	1017–3.670		1.848	0.826–4.134
Age	< 35 years	242	79.9	49	83.1	193	79.1	0.497	1.295	0.614–2.732			
	≥ 35 years	61	20.1	10	16.9	51	20.9		Ref.				
Education	≤ 8 years	126	41.9	24	40.7	102	42.1	0.837	Ref.				
	> 8 years	175	58.1	35	59.3	140	57.9		1.063	0.596–1.895			
Race	White	156	51.8	27	45.8	129	53.3	0.298	Ref.				
	Other	145	48.2	32	54.2	113	46.7		1.353	0.764–2.395			
Work outside the home	Yes	139	46.3	23	39	116	48.1	0.207	Ref.				
	Not	161	53.7	36	61	125	51.9		1.453	0.812–2.597			
Person per room	<1	125	45.3	21	41.2	104	46.2	0.513	Ref.				
	≥1	151	54.7	30	58.8	121	53.8		1.228	0.663–2.274			
Knowledge of ZIKV transmission		282		53		229							
	Insect vector	236	83.7	44	83.0	192	83.8	0.884	0.942	0.424–2.094			
	Perinatal	116	41.1	18	34.0	98	42.8	0.239	0.687	0.368–1.285			
	Sexual intercourse	121	42.9	25	47.2	96	41.9	0.487	1.237	0.679–2.253			
	None	40	14.2	9	17	31	13.5	0.517	1.306	0.581–2.939			
Source of knowledge		222		44		178							
	Internet	68	30.6	12	27.3	56	31.5	0.589	0.817	0.392–1.704			
	Television	131	59	22	50.0	109	61.2	0.175	0.633	0.326–1.229	0.049*	0.493	0.244–0.997
	Health professional	149	67.1	32	72.7	117	65.7	0.376	1.390	0.669–2.891			
Primigravid	Yes	92	32.7	23	42.6	69	30.4	0.086	1.699	0.924–3.124	0.203	1.608	0.774–3.344
	Not	189	67.3	31	57.4	158	69.6		Ref.			Ref.	

*$p < 0.05$; CI: Confidence interval; aOR: adjusted Odds Ratio.

Table S3. Univariate and multivariate logistic regression analysis of associations between variables and ZIKV infection.

**Variables**		**All subjects**		**ZIKV**				***p*–value**	**OR**	**[CI 95%]**	***p*–value**	**aOR**	**[CI 95%]**
				Positive		Negative							
		n	%	n	%	n	%						
Marital status	Stable union	247	78.7	28	82.4	219	78.2	0.578	Ref.				
	Not stable partner	67	21.3	6	17.6	61	21.8		1.300	0.515–3.282			
Age	< 35 years	253	80.3	29	85.3	224	79.7	0.440	Ref.				
	≥ 35 years	62	19.7	5	14.7	57	20.3		1.476	0.547–3.982			
Education	≤ 8 years	131	41.9	15	44.1	116	41.6	0.777	Ref.				
	> 8 years	182	58.1	19	55.9	163	58.4		1.109	0.541–2.274			
Race	White	163	52.1	19	55.9	144	51.6	0.638	Ref.				
	Other	150	47.9	15	44.1	135	48.4		1.187	0.580–2.431			
Work outside the home	Yes	144	46.2	16	47.1	128	46.0	0.911	Ref.				
	Not	168	53.8	18	52.9	150	54.0		1.042	0.510–2.126			
Person per room	<1	128	44.6	13	39.4	115	45.3	0.523	1.273	0.607–2.670			
	≥1	159	55.4	20	60.6	139	54.7		Ref.				
Insect repellent usage	Yes	167	53.0	22	64.7	145	51.6	0.148	Ref.		0.175	Ref.	
	Not	148	47.0	12	35.3	136	48.4		1.720	0.819–3.609		1.754	0.779–3.949
Condom usage	Yes	59	19.5	6	17.6	53	19.7		1.145	0.451–2.907			
	Not	244	80.5	28	82.4	216	80.3	0.775	Ref.				
Knowledge of ZIKV transmission		290		29		261							
	Insect vector	243	83.8	27	93.1	216	82.8	0.190	0.356	0.082–1.549	0.315	0.461	0.102–2.090
	Perinatal	122	42.1	14	48.3	108	41.4	0.475	0.756	0.351–1.632			
	Sexual intercourse	124	42.8	17	58.6	107	41.0	0.069	0.490	0.225–1.069	0.192	0.585	0.262–1.308
	None	41	14.1	2	6.9	39	14.9	0.397	2.372	0.542–10.378			
Prophylactic orientations	Yes	229	79.0	24	82.8	205	78.5	0.597	Ref.				
	Not	61	21.0	5	17.2	56	21.5		1.311	0.479–3.592			
Source of knowledge		229		24		205							
	Internet	70	30.6	10	41.7	60	29.3	0.212	0.579	0.244–1.377			

	Television	136	59.4	17	70.8	119	58.0	0.228	0.570	0.226–1.434
	Health professional	152	66.4	17	70.8	135	65.9	0.625	0.794	0.314–2.005
Primigravid	Yes	94	32.4	14	41.2	80	31.3		Ref.	
	Not	196	67.6	20	58.8	176	68.8	0.245	1.540	0.740–3.203

CI: Confidence interval; aOR: adjusted Odds Ratio.
